# A Web-Based Cognitive Bias Modification Intervention (Re-train Your Brain) for Emerging Adults With Co-occurring Social Anxiety and Hazardous Alcohol Use: Protocol for a Multiarm Randomized Controlled Pilot Trial

**DOI:** 10.2196/28667

**Published:** 2021-07-07

**Authors:** Katrina Prior, Elske Salemink, Reinout W Wiers, Bethany A Teachman, Monique Piggott, Nicola C Newton, Maree Teesson, Andrew J Baillie, Victoria Manning, Lauren F McLellan, Alison Mahoney, Lexine A Stapinski

**Affiliations:** 1 The Matilda Centre for Research in Mental Health and Substance Use The University of Sydney Sydney Australia; 2 Experimental Psychopathology Lab Department of Clinical Psychology Utrecht University Utrecht Netherlands; 3 Addiction Development and Psychopathology (ADAPT) Lab Department of Psychology University of Amsterdam Amsterdam Netherlands; 4 Center for Urban Mental Health University of Amsterdam Amsterdam Netherlands; 5 Department of Psychology School of Arts and Sciences University of Virginia Virginia, VA United States; 6 Sydney School of Health Sciences The University of Sydney Sydney Australia; 7 Eastern Health Clinical School Faculty of Medicine, Nursing & Health Sciences Monash University Melbourne Australia; 8 Centre for Emotional Health Department of Psychology Macquarie University Sydney Australia; 9 Clinical Research Unit for Anxiety and Depression St Vincent's Public Hospital Sydney Australia

**Keywords:** alcohol, anxiety, cognitive bias modification, interpretation bias, approach bias, emerging adults

## Abstract

**Background:**

Alcohol use and anxiety disorders commonly co-occur, resulting in a more severe clinical presentation and poorer response to treatment. Research has shown that approach bias modification (ApBM) and interpretation bias modification (IBM) cognitive retraining interventions can be efficacious adjunctive treatments that improve outcomes for alcohol use and social anxiety, respectively. However, the acceptability, feasibility, and clinical utility of combining ApBM and IBM programs to optimize treatments among comorbid samples are unknown. It is also unclear whether integrating ApBM and IBM *within* each training session or alternating them *between* each session is more acceptable and efficacious.

**Objective:**

This paper describes the protocol for a randomized controlled pilot trial investigating the feasibility, acceptability, and preliminary efficacy of the *Re-train Your Brain* intervention—an adjunct web-based ApBM+IBM program—among a clinical sample of emerging adults with hazardous alcohol use and social anxiety.

**Methods:**

The study involves a three-arm randomized controlled pilot trial in which treatment-seeking emerging adults (18-30 years) with co-occurring hazardous alcohol use and social anxiety will be individually randomized to receive the *Re-train Your Brain integrated* program, delivered with 10 biweekly sessions focusing on *both* social anxiety and alcohol each week, plus treatment as usual (TAU; ie, the model of care provided in accordance with standard practice at their service; n=30); the *Re-train Your Brain alternating* program, delivered with 10 biweekly sessions focusing on social anxiety one week and alcohol the next week, plus TAU (n=30); or TAU only (n=30). Primary outcomes include feasibility (uptake, follow-up rates, treatment adherence, attrition, and adverse events) and acceptability (system usability, client satisfaction, user experience, and training format preference). Secondary efficacy outcomes include changes in alcohol approach and interpretation biases, social anxiety, and alcohol use (eg, drinks per day, binge drinking, drinking motives, severity of dependence, and cravings). The primary end point will be posttreatment (6 weeks postbaseline), with a secondary end point at 3 months postbaseline. Descriptive statistics will be conducted for primary outcomes, whereas intention-to-treat, multilevel mixed effects analysis for repeated measures will be performed for secondary outcomes.

**Results:**

This study is funded from 2019 to 2023 by Australian Rotary Health. Recruitment is expected to be completed by mid-2022 to late 2022, with follow-ups completed by early 2023.

**Conclusions:**

This study will be the first to evaluate whether an ApBM+IBM program is acceptable to treatment-seeking, emerging adults and whether it can be feasibly delivered via the web, in settings where it will ultimately be used (eg, at home). The findings will broaden our understanding of the types of programs that emerging adults will engage with and whether the program may be an efficacious treatment option for this comorbidity.

**Trial Registration:**

Australian New Zealand Clinical Trials Registry ACTRN12620001273976; https://www.anzctr.org.au/Trial/Registration/TrialReview.aspx?id=364131

**International Registered Report Identifier (IRRID):**

PRR1-10.2196/28667

## Introduction

### Background

Social anxiety and alcohol use disorders are highly prevalent [1] and frequently co-occur [2,3]. When they are comorbid with one another, the presenting symptoms tend to be more severe and associated with greater functional impairment than either disorder in isolation [4,5]. This is likely because of the existence of a bidirectional, self-perpetuating cycle, whereby ongoing interactions between the disorders serve to maintain or exacerbate symptoms of both conditions [4,6,7]. The co-occurrence of social anxiety and alcohol use disorders can also interfere with treatment and recovery, with evidence from clinical trials showing that standard single-disorder treatments are less effective for people with comorbid anxiety and alcohol use [4,8-11].

Given the frequent co-occurrence of these disorders and the additional complications that this comorbidity confers on the individual (eg, physical and psychological health, relationships, work, and education) and society (eg, social and economic costs) [4,5,12], it is essential to understand and modify factors that contribute to the maintenance of these conditions. One such factor is implicit or automatically activated cognitive biases. Although there are a variety of implicit cognitive biases associated with social anxiety and alcohol use disorders, two of the most common are alcohol *approach biases* (ie, the tendency for alcohol cues to induce an automatic and habitual approach action [13,14]) and *interpretation biases* (ie, the tendency to interpret ambiguous stimuli, scenarios, and events in a negative manner [15-17]). Alcohol approach biases have been shown to contribute to heavy drinking and predict future alcohol use among adults with an alcohol use disorder [14] and have also been associated with relapse to alcohol following treatment [18,19]. Interpretation biases have been linked to the development of anxiety symptoms and disorders, and the maintenance and severity of these conditions [20,21].

Over the past two decades, several computer-based cognitive training paradigms, known as cognitive bias modification (CBM), have been developed to reduce symptoms by modifying a range of maladaptive implicit biases, including alcohol approach and anxiety-related interpretation biases. *Approach bias modification* (ApBM) is one of the most common types of CBM programs trialed as an adjunctive treatment for alcohol use disorders. It seeks to train adaptive alcohol-avoidance tendencies by getting an individual to repeatedly push away images of alcohol shown on a computer screen. *Interpretation bias modification* (IBM) is one of the dominant CBM programs for ameliorating anxiety symptoms, particularly social anxiety. IBM directly targets negative interpretation biases by repeatedly presenting individuals with emotionally ambiguous social scenarios and training them to resolve the uncertainty in a positive or neutral (vs threatening) way [22]. Several large randomized controlled trials (RCTs) among clinical samples show particularly promising findings for ApBM and IBM interventions, especially in instances where they are added as an adjunct to standard evidence-based treatments, such as cognitive behavioral therapy (as described in detail later) [23-28]. It is argued that this is likely because clients in treatment are typically motivated to change their thoughts and behaviors, which may be a prerequisite for successful training effects [29]. By adding CBM on top of conventional treatment approaches, treatment not only is able to target conscious, deliberate, and explicit negative thinking styles and behavioral responses but can also target unhealthy habitual and implicit processes [30-32]. Thus, a combination of these complementary approaches further aids in the effectiveness of treatment on clinical outcomes. In contrast, there has been mixed empirical support for the efficacy of ApBM and IBM programs as stand-alone interventions among nonclinical samples (eg, heavy drinking university students) [29,33,34]. Meta-analyses that synthesize findings on efficacy from a mixture of clinical and nonclinical studies have produced similar inconclusive findings [34,35], perhaps as a result of the substantial heterogeneity in study samples, designs, and methodologies [29,36].

Several large RCTs [23-26] and reviews [29,37,38] have provided evidence in support of the beneficial effects of ApBM when delivered to clinical samples in conjunction with standard treatments. Notably, 3 studies in Germany showed that the provision of 4-12 sessions of ApBM alongside treatment as usual (TAU; eg, inpatient alcohol use treatment or abstinence-oriented cognitive behavioral therapy) to alcohol-dependent adults was associated with significantly lower alcohol consumption and 7%-13% reduced rates of relapse one year later compared with patients who received TAU plus a sham placebo training or TAU only [23,24,26]. One of these studies reported that the change in alcohol approach biases mediated the change in relapse rates and a stronger approach bias at baseline moderated ApBM effects [24], whereas another was unable to replicate these effects [26]. Furthermore, a recent multisite RCT conducted in Australia reported significant improvements in abstinence rates among those given four sessions of ApBM during inpatient detoxification (54%), relative to sham control training (42%) [39]. A significant reduction in approach bias was observed among those in ApBM but not in the sham-training condition after training. Overall, ApBM has shown fairly consistent promising effects in clinical contexts and has since been added to German and (forthcoming) Australian guidelines for treating alcohol-related disorders [40,41].

Clinical studies investigating the efficacy of IBM have shown that training can promote the development of a positive interpretive bias in socially anxious populations and that these modifications are associated with reductions in social anxiety symptoms [27,28]. Although there are fewer clinical RCTs in number relative to ApBM, one study in the United States found that adults diagnosed with generalized social anxiety disorder who received 12 sessions of IBM experienced significantly greater reductions in negative interpretations of ambiguous scenarios, self-reported and clinician-rated social anxiety symptoms, and rates of social anxiety disorder diagnosis (65% vs 13%) from pre- to postassessment, relative to a sham control-training condition. Moreover, the effects on social anxiety were sustained at the 3-month follow-up [27]. Similarly, a pilot study found that the provision of four IBM sessions combined with CBM for attentional biases to an outpatient sample diagnosed with social or generalized anxiety disorder was associated with significant reductions in both types of cognitive bias and state and trait anxiety [28]. Studies investigating the mechanism of change for these effects have reported that negative interpretation biases mediated the relationship between the training group and improvement in social anxiety symptoms [27]. These findings are also supported by a recent review of meta-analyses and a systematic review and meta-analysis of CBM programs for anxiety, which both concluded that single- or multisession IBM training among clinically diagnosed and subclinical samples can significantly reduce threat-related interpretation biases [42] and reduce anxiety levels compared with a sham training or waitlist control condition [43].

A limitation to the clinical utility of ApBM and IBM interventions to date is that they have predominantly been restricted to the confines of a laboratory or clinic [44]. Despite this, internet delivery is a promising option for increasing the scalability and sustainability of these interventions. A handful of published studies evaluating the effectiveness of web-based ApBM and IBM have indicated that there is promise in this mode of delivery; however, additional studies are required to make more definitive conclusions regarding their efficacy. For instance, two studies in Europe provided evidence that web-based IBM training can significantly shift interpretations from negative to positive [45,46], with one study showing clinically significant improvement in social anxiety symptoms (eg, 48% of participants no longer met criteria for social anxiety disorder after eight IBM sessions) [46]. Another study found that four sessions of web-based ApBM among nontreatment-seeking problem drinkers were associated with improved drinking outcomes across three active intervention groups; however, this effect was also evident in the sham control-training group [47]. The authors highlight that despite their null findings, integrating web-based ApBM with more traditional cognitive and motivational interventions may be key to improving results and call for further research that combines these supplementary treatments. It is apparent that there is a need for more studies of web-based CBM programs (particularly ApBM) with clinical populations where there is a motivation to change their thoughts and/or behaviors.

Overall, although the evidence base for the efficacy of laboratory- and web-based ApBM and IBM is accumulating for adults with singular disorders, their efficacy when delivered to people with co-occurring social anxiety and alcohol use problems remains largely unknown. To the authors’ knowledge, only one RCT exists that examines the efficacy of an attention bias–focused CBM program among a socially anxious, alcohol-dependent adult sample (N=86) [48]. The findings indicated that there were significant reductions in attentional biases, alcohol use disorder, and social anxiety symptoms in both the intervention and control groups; however, no significant between-group differences were identified for any symptom measures. Given the interconnections between anxiety and alcohol use problems and the likely coexistence of approach and interpretation biases, a promising avenue that has not been explored is the potential of combining existing effective ApBM and IBM cognitive retraining protocols to optimize standard treatments among comorbid anxiety-alcohol samples. Moreover, given the peak onset and disability associated with anxiety and alcohol use disorders occurs between adolescence and early adulthood [49,50], the provision of a comorbidity-focused ApBM+IBM training program at an earlier age (and earlier in the course of their disorder) represents a promising opportunity to intervene before problems become chronic and entrenched in adulthood.

Addressing these gaps in the literature, the research team codeveloped a hybrid, web-based ApBM+IBM for emerging adults with co-occurring hazardous alcohol use and social anxiety (*Re-train Your Brain*), as a supplement to TAU for anxiety and/or alcohol use. As trial repetition, boredom, and disengagement are serious concerns for CBM training [28], it deemed was important to assess attitudes toward this treatment and adapt the program where necessary. In line with this, a study was conducted to evaluate the acceptability of a beta version of the *Re-train Your Brain* program according to the perspectives of clinicians and emerging adults with hazardous drinking and heightened social anxiety [51]. The results indicated that the ApBM+IBM intervention was an acceptable adjunct to traditional evidence-based treatments with potential clinical utility, a finding that mirrors previous CBM acceptability studies [52,53]. To enhance engagement, clinical utility, and intrinsic motivation to complete the training, clinicians and emerging adults suggested that the program should include a psychoeducational and motivation enhancement-type module before the first training session. In light of the aforementioned issues surrounding boredom and disengagement from CBM programs, it was considered possible that the format in which the ApBM+IBM program is delivered might influence engagement and outcomes. For instance, one delivery option could be to integrate the ApBM and IBM components into each training session (50:50 ratio) to provide variation in tasks *within* each training session and reduce boredom. This may, in turn, result in better retention, engagement, and outcomes because of a shorter time being spent completing each repetitious task. An integrated variation might also be especially beneficial because it gives people practice shifting multiple cognitive processes close in time, which likely better mimics the cognitive flexibility needed in daily life (where a cue may require both healthy alcohol-avoidance reactions and healthy interpretation bias in a short space of time) as compared with more temporally distant application of modified cognitive processes. It is equally possible, however, that doing shorter bursts of the two types of training within each session may water down any potential clinical effects. Another option could be to alternate the ApBM and IBM training *between* sessions, thereby providing full-length sessions of each type of training, although fewer in number to achieve the same treatment dose. Clinicians and emerging adults were asked to rate the format they perceived would be most preferable to end users of the program: (1) an *integrated* program that combines shortened versions of both ApBM and IBM within each training session or (2) an *alternating* program that provides ApBM in one training session and IBM in the next, in an alternating pattern. The results indicated that there was no consistent preference for the program format (8/15, 53% vs 7/15, 47% for integrated and alternating, respectively), and further research is required to better understand the impact of training format on acceptability and efficacy outcomes. Overall, the beta *Re-train Your Brain* ApBM+IBM program was well received, and obtaining feedback from service providers and emerging adults was critical to ensuring that the program was age-appropriate, engaging, and potentially useful for end users. However, to date, no research has been conducted to evaluate the feasibility of delivering this program, whether it is deemed acceptable by people who complete the training, and how efficacious it is in reducing the targeted cognitive biases, hazardous drinking, and social anxiety symptoms.

### Aims

This paper describes the study protocol for a randomized controlled pilot trial investigating the feasibility, acceptability, and preliminary efficacy of the *Re-train Your Brain* intervention—a web-based hybrid ApBM+IBM program—as an adjunct to TAU for anxiety and/or alcohol use, compared with TAU only, among emerging adults with co-occurring hazardous alcohol use and social anxiety disorder symptoms. The *Re-train Your Brain* program will be delivered in two formats (integrated or alternating) to ascertain which is preferred by emerging adults and whether one shows more promising signs of efficacy.

It is hypothesized that the integrated and alternating *Re-train Your Brain* ApBM+IBM programs will both be feasible to implement and deemed acceptable by emerging adults. It is hypothesized that the integrated format will be rated as more acceptable and engaging than the alternating format because of the variation of tasks *within* each session (ie, less time will be spent on each task within each session, which may reduce boredom and disengagement often experienced because of the repetitive nature of each task). Although this is a pilot trial that is not powered to detect clinically significant group-by-time interaction effects, based on previous research, it is likely that both the integrated and alternating *Re-train Your Brain* programs will result in trend improvements in cognitive biases, anxiety, and alcohol-related outcomes, relative to the control condition. It is further hypothesized that greater effect sizes will be observed in the integrated *Re-train Your Brain* intervention group than in the alternating intervention group because of greater levels of engagement with the program. The results will be used to inform the design and power analysis of a future definitive trial.

## Methods

### Setting and Trial Design

**Figure 1:**
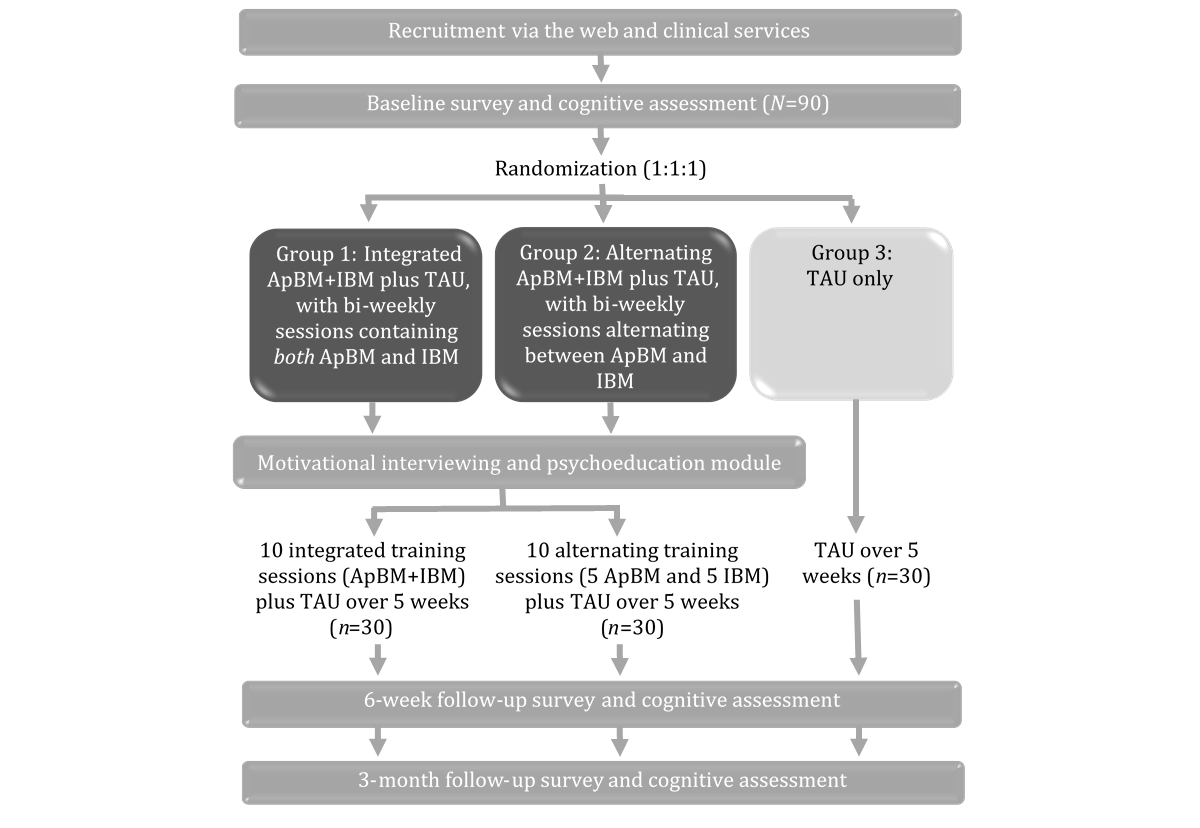
Study design for the *Re-train Your Brain* pilot trial. ApBM: Approach Bias Modification; IBM: Interpretation Bias Modification; TAU: treatment as usual.

The study will be conducted nationally across Australia and will involve a three-arm randomized controlled pilot trial in which eligible participants will be individually randomized to receive (1) the *integrated Re-train Your Brain* program, delivered with 10 biweekly sessions focusing on *both* social anxiety and alcohol each training (50:50 ratio) plus TAU for anxiety and/or alcohol use (ie, the model of care provided in accordance with standard practice at their service); (2) the *alternating Re-train Your Brain* program, delivered with 10 biweekly sessions focusing on social anxiety in one training and alcohol in the next training in an alternating pattern plus TAU; or (3) TAU only. The treatment *dose* in terms of total intervention time will be the same for the two intervention groups (groups 1 and 2). The primary end point will be the posttreatment assessment, conducted at 6 weeks following baseline, with a secondary end point at 3 months postbaseline. The study design is shown in Figure 1.

### Ethics Approval and Registration

The *Re-train Your Brain* pilot trial was prospectively registered with the Australian New Zealand Clinical Trials Registry (ACTRN12620001273976) and received ethical approval from the University of Sydney Human Research Ethics Committee (#2020/135).

### Participants

#### Participant Recruitment

Young Australians (n=90) aged 18 to 30 years with harmful alcohol use and heightened social anxiety symptoms who are currently receiving psychological treatment will be recruited into the study via an array of advertising methods, including clinician referral, poster, and flyer advertisements (eg, in clinical services and universities), and online advertising via platforms, such as Facebook, Twitter, and Google AdWords.

#### Inclusion and Exclusion Criteria

To be eligible, participants must (1) be Australians aged 18-30 years; (2) be currently reporting hazardous or harmful alcohol use, that is, a score of ≥8 on the Alcohol Use Disorder Identification Test [54]; (3) be currently experiencing at least mild symptoms of social anxiety, that is, a score of ≥7 on the Social Interaction Anxiety Scale-Short Form or ≥2 on the Social Phobia Scale-Short Form [55]; (4) have access to the internet via a laptop or PC and have a mouse-operable computer; (5) be currently receiving psychological treatment from a health professional for anxiety and/or alcohol use problems, for example, psychologist, psychiatrist, mental health, or alcohol or other drug counselor; and (6) be willing to complete all 10 ApBM+IBM sessions, if allocated to one of the active intervention groups.

Individuals will be excluded because of the following reasons: inability or unwillingness to provide contact information (ie, phone and email); insufficient English literacy; active symptoms of psychosis, that is, a score of ≥3 on the Psychosis Screening Questionnaire [56]; self-reported history of neurological disease or head injury with a loss of consciousness exceeding 30 minutes; self-reported intellectual disability or cognitive impairment; and eyesight not normal or corrected to normal.

### Measures

#### Overview

Table 1 summarizes the schedule of assessments (including measures used for primary and secondary outcomes and potential moderators or covariates) and procedures over the study period.

**Table 1:** Schedule of assessments and procedures over the study period.

Assessments				Study period																	
				Enrollment		Preallocation; *t*_1_ (baseline)		Intervention week											Postallocation		
								1		2		3		4		5		*t*_2_ (6 weeks postbaseline)			*t*_2_ (3 months postbaseline)
**Enrollment**																					
	Informed consent			✓^a^																	
	Eligibility survey			✓																	
	Baseline survey					✓															
	Cognitive assessments					✓												✓			✓
	Randomization					✓															
**Interventions**																					
	Group 1: Integrated ApBM^b^+IBM^c^ plus TAU^d^							✓		✓		✓		✓		✓					
	Group 2: Alternating ApBM+IBM plus TAU							✓		✓		✓		✓		✓					
	Group 3: TAU (routine anxiety or alcohol treatment)							✓		✓		✓		✓		✓					
**Assessments or measures**																					
	**Primary outcomes**																				
		Treatment feasibility questions															✓				
		Acceptability questions															✓				
		System Usability Scale															✓				
		Client Satisfaction Questionnaire															✓				
		Treatment feedback questions															✓				
		Postuser experience questions (intervention groups)					✓		✓		✓		✓		✓						
	**Secondary outcomes and covariates or moderator variables**																				
		Approach Avoidance Task			✓												✓			✓	
		Interpretation Recognition Task			✓												✓			✓	
		Comorbid Interpretation and Expectancy Biases			✓												✓			✓	
		Social Phobia Scale and Social Interaction Anxiety Scale-Short Forms	✓														✓			✓	
		Patient Health Questionnaire-4			✓												✓			✓	
		Alcohol Use Disorder Identification Test	✓														✓			✓	
		Timeline Follow Back			✓												✓			✓	
		Alcohol Craving Questionnaire–Short Form–Revised			✓												✓			✓	
		Severity of Alcohol Dependence Questionnaire			✓												✓			✓	
		Drinking Motives Questionnaire-Revised			✓												✓			✓	
		Weekly Social Anxiety and Alcohol Symptom questions					✓		✓		✓		✓		✓						
		Readiness to Change Ruler			✓												✓			✓	
		University of Rhode Island Change Assessment			✓																
		Psychological and pharmacological treatment			✓												✓			✓	

^a^Denotes which assessments and/or measures were conducted at each time point.

^b^ApBM: approach bias modification.

^c^IBM: interpretation bias modification.

^d^TAU: treatment as usual.

#### Primary Outcome Measures

##### Feasibility

The feasibility of the program will be assessed according to the percentage of successfully recruited participants who agree to participate (ie, uptake), commence training, and decline participation. At postintervention, feasibility will be measured by the number of sessions completed; reporting of adverse events via spontaneous reports in open-feedback questions or to the study team or deterioration of social anxiety or alcohol use symptoms (see Multimedia Appendix 1 [54,55,57-66] for full details) [57,58]; and the proportion of participants who (1) complete the 10-session protocol (as a proportion of those who commence at least one session of training, ie, treatment adherence or compliance), (2) complete the mean optimum number of six sessions, based on ApBM research [67], and (3) drop out before training is completed. Survey or cognitive assessment follow-up rates will also be recorded as a measure of the feasibility of the RCT methodology at the 6-week and 3-month time points.

##### Acceptability

Measures of acceptability and usability of the program will be assessed at postintervention (6-week postbaseline). *The usability of the program* will be assessed using the 10-item System Usability Scale [68]. Items are rated on a 5-point Likert scale ranging from 1 (*strongly disagree*) to 5 (*strongly agree*). The scores for each item are converted to a new number, ranging from 0 to 4. A total score is then computed by summing the converted item scores and multiplying this by 2.5, giving a range of 0 to 100. Higher values denote greater usability and higher satisfaction with the program. Cut-off scores using a *school grade analogue* have recently been suggested for interpreting the scores (F=0-51.7; D=51.8-62.6; C=62.7-72.5; B=72.6-78.8; and A=78.9-100), with a score over 68 being considered above average [69]. *Satisfaction* with the program will be measured by Client Satisfaction Questionnaire-8 [70]. Items are rated on a 4-point Likert scale from 1 to 4, with total scores ranging from 8 to 32. Higher scores indicate greater client satisfaction. Acceptability will also be assessed by 13 acceptability items (eg, how user-friendly, simple to use, logical, and engaging the tasks were), rated using a 5-point Likert scale from 0 (*not at all*) to 4 (*very*). The survey will also contain several open-ended questions about the most or least helpful and enjoyable features of the program. To determine *which intervention delivery model is preferred*, participants in the active intervention groups will complete four user experience questions after completing each of the *Re-train Your Brain* sessions. The four items will assess their motivation while training, how much they enjoyed the training, how much they liked the delivery format, and whether they found the training simple and user-friendly, using a 5-point Likert scale (from 0 *not at all* to 4 *extremely*).

#### Secondary Outcome Measures

##### Overview

The following measures will be assessed at baseline, postintervention, and 3 months postbaseline (a more detailed description and interpretation of scores are given in Multimedia Appendix 1).

##### Cognitive Biases

*Alcohol approach biases* will be assessed using the Alcohol Approach Task [13]. Participants are instructed to pull or push a computer mouse (in place of a joystick, which is typically used in laboratory-based studies) in response to an irrelevant feature of images (ie, the orientation as portrait or landscape) shown on a computer screen while ignoring the content of the pictures. Two categories of pictures are used: 20 alcoholic beverages and 20 color- and shape-matched nonalcoholic beverages. Each picture appears one by one in both landscape and portrait formats in a quasi-random order (maximum 3 consecutive pictures of the same category or format). Contingent upon a pull or push movement, the picture zooms in (becomes larger on the screen to generate the subjective experience of an approach behavior) or zooms out (becomes smaller on the screen to give a sense of avoidance behavior). A correct response is followed by feedback, as indicated by a green ✓on the screen, whereas an incorrect response is followed with error feedback, as indicated by a red X. The task begins with 5 practice trials showing empty rectangular frames in a landscape or portrait format. Format movement assignments are counterbalanced (half of the participants pull pictures that came in landscape format and push portrait pictures, and half of the participants received the opposite instruction). The images have been selected to represent the beverage type and brands most commonly consumed by this population, as documented in a recent acceptability study [51]. Each image is repeated twice, for a total of 80 trials.

*Social anxiety interpretation biases* will be measured using the Interpretation Recognition Task [22,71]. The Interpretation Recognition Task contains two parts: an encoding phase and a recognition test phase. During the encoding phase, participants are asked to read a set of 10 ambiguous social scenarios presented on a computer screen. Each scenario consists of a title and three short sentences that are ambiguous in terms of valence. In the final sentence, a word fragment is presented. After reading a scenario, participants are asked to complete the word fragment as quickly as possible by pressing the spacebar when they know what the word is. They then press the key corresponding to the first missing letter. Next, the participant is asked to answer yes or no to a question that measures comprehension of the story, also followed by feedback (ie, *correct* or *wrong*). An example of the encoding phase is provided as follows:

(Title): *The evening class.*

(Scenario): You’ve just started going to an evening class. The instructor asks a question and no one in the group volunteers an answer, so he looks directly at you. You answer the question, aware of how your voice must sound to the...

(Word fragment): oth-rs

(Correct word): others

(Comprehension question): Have you been going to the evening class for a long time?

(Response): No

(Feedback): ✓ Correct answer

In the recognition test phase, participants are presented with the identifying titles of each ambiguous scenario, together with four interpretations of each of the scenarios, presented one at a time. Participants are asked to rate each summary statement independently for their similarity in meaning to the original scenario on a 4-point Likert scale from 1 (*very different in meaning*) to 4 (*very similar in meaning*). The four summary statements always contain one valid, positively valanced interpretation (positive target); one valid, negatively valanced interpretation (negative target); one positively valanced statement that was not a possible interpretation of the original scenario in that it did not correspond to the previously presented ambiguity (positive foil); and one negatively valanced statement that was not a possible interpretation of the original scenario (negative foil). Foils are included to assess any wider priming effects, indicating a potential response bias for endorsing any information of a certain emotional valence. Examples of the summary statements that would be provided for the aforementioned scenario are shown as follows:

1. Positive target: You answer the question, aware of others listening attentively.
2. Negative target: You answer the question, aware of how unsteady your voice sounds.
3. Positive foil: You answer the question and then realize what a good answer it is.
4. Negative foil: You answer the question but realize that you have made a mistake.

*Interpretation and expectancy biases for co-occurring social anxiety and alcohol use* will be assessed by the self-report Comorbid Social Anxiety and Alcohol Interpretation Bias task [59]. Participants are presented with a set of eight ambiguous social scenarios, followed by three possible explanations for the situation. Participants will rate the degree to which each of the explanations would likely be true if they were in that situation (0 *not at all likely* to 8 *extremely*).

##### Alcohol Use

*Alcohol consumption* (average drinks per day) and frequency of binge drinking days (>5 standard drinks per drinking day) in the past month will be assessed through a computerized version of the Timeline Followback Procedure [60-62]. *Hazardous alcohol use* will be assessed using the 10-item Alcohol Use Disorder Identification Test [54]. *Severity of alcohol dependence* will be assessed using the 20-item Severity of Alcohol Dependence Questionnaire [63]. *Alcohol cravings* will be assessed using the 12-item Severity of Alcohol Craving Questionnaire–Short Form–Revised. *Motives for alcohol use* in the past month will be assessed through the 28-item Drinking Motives Questionnaire-Revised [64]. To assess *alcohol use* throughout the intervention period, all groups will be asked to complete the Timeline Followback twice per week [60-62].

##### Anxiety

*Symptoms of social anxiety* will be assessed using the short forms of the Social Interaction Anxiety Scale and the Social Phobia Scale [55]. *Symptoms of depression and anxiety* will be assessed using the 4-item Patient Health Questionnaire-4 [65]. To assess changes in *social anxiety symptoms* throughout the intervention period, all groups will be asked to complete the Social Phobia Weekly Summary Scale [66] biweekly.

##### Covariates and Additional Variables

*Sociodemographic characteristics* will include age, sex, gender, education, employment, country of birth, and primary mental health or substance use concerns. Participants will also be asked about any *psychological and pharmacological treatment* received in the past 3 months (full details given in Multimedia Appendix 1). *Readiness and motivation to change* will be assessed via a readiness ruler (eg, on a scale of 1-10, how ready are you to change your anxiety or drinking) and the University of Rhode Island Change Assessment [72]. The *frequency of other drug use* (cannabis, nonprescribed benzodiazepines, and psychostimulants) will be assessed by the National Institute on Drug Abuse quick screen [73], and *sleep disturbance* over the past month will be assessed using the Pittsburgh Sleep Quality Index [74].

### Intervention and Control Groups

#### Re-train Your Brain ApBM+IBM Intervention Groups

##### Overview

Both the integrated and alternating Re-*t*rain Your Brain interventions contain ApBM and IBM components, delivered in addition to TAU. Participants in both groups will be asked to complete 10 training sessions over a 5-week period. Two sessions will be available to complete each week, and participants will be given 1-week flexibility to complete all the 10 training sessions. Each training session will be of approximately 20 minutes. In addition, as per previous trials [52], before the commencement of the first training session, participants in both intervention groups will receive an online psychoeducational and motivational interviewing–based module, which was adapted from a previous efficacious online program for anxiety and alcohol use [75]. The module provides psychoeducation about anxiety and alcohol use, the interrelationship between these problems, the existence of automatically activated cognitive biases, and the importance of changing these biases. It also explores participants’ reasons for change, and aims to increase intrinsic motivation for (and decrease ambivalence about) change, promotes autonomy and *change talk*, helps participants set goals for what they hope to achieve by completing the training, enhances motivation to train, and harnesses the individual’s capacity for change. The ApBM and IBM components of the integrated and alternating training programs are described later.

##### Alcohol ApBM Component

**Figure 2:**
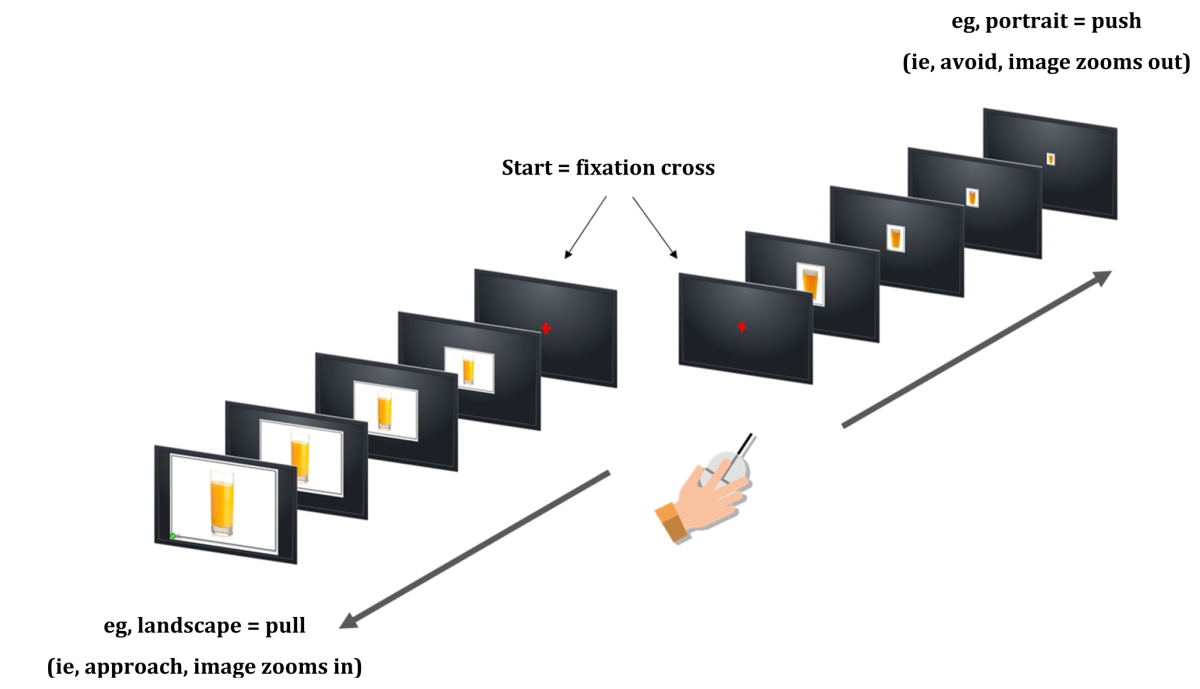
Example of Approach Bias Modification scenario to illustrate the training procedures.

ApBM is a modified training version of the assessment Alcohol Approach Task, in which participants pull or push a computer mouse in response to the orientation of images (containing alcoholic or nonalcoholic beverages), which zooms the image in or out (Figure 2). For ApBM (unlike the Alcohol Approach Task), 95% of the orientations used to implicitly train avoidance behaviors will contain images of alcoholic beverages, whereas the remaining 5% of frames will contain images of nonalcoholic beverages (and vice versa for orientations used to train approach behaviors). The required push-pull movements are counterbalanced so that half of the participants pull pictures that come in landscape format and push pictures in portrait format, whereas the other half receive the opposite instruction. Participants receive feedback in the form of a green tick or red cross presented on-screen, with a corresponding smiley or sad face emoji. The presentation is repeated if the response is incorrect. To increase motivation, points are awarded for each correct push-pull movement (+1). To familiarize participants with the task requirements, a brief practice round involving five empty rectangular frames in landscape or portrait format will be provided. The alcoholic and nonalcoholic images used in this study were selected to represent the beverage types and brands most commonly consumed by this population, as documented in a recent acceptability study [51].

##### Anxiety IBM Program Component

**Figure 3:**
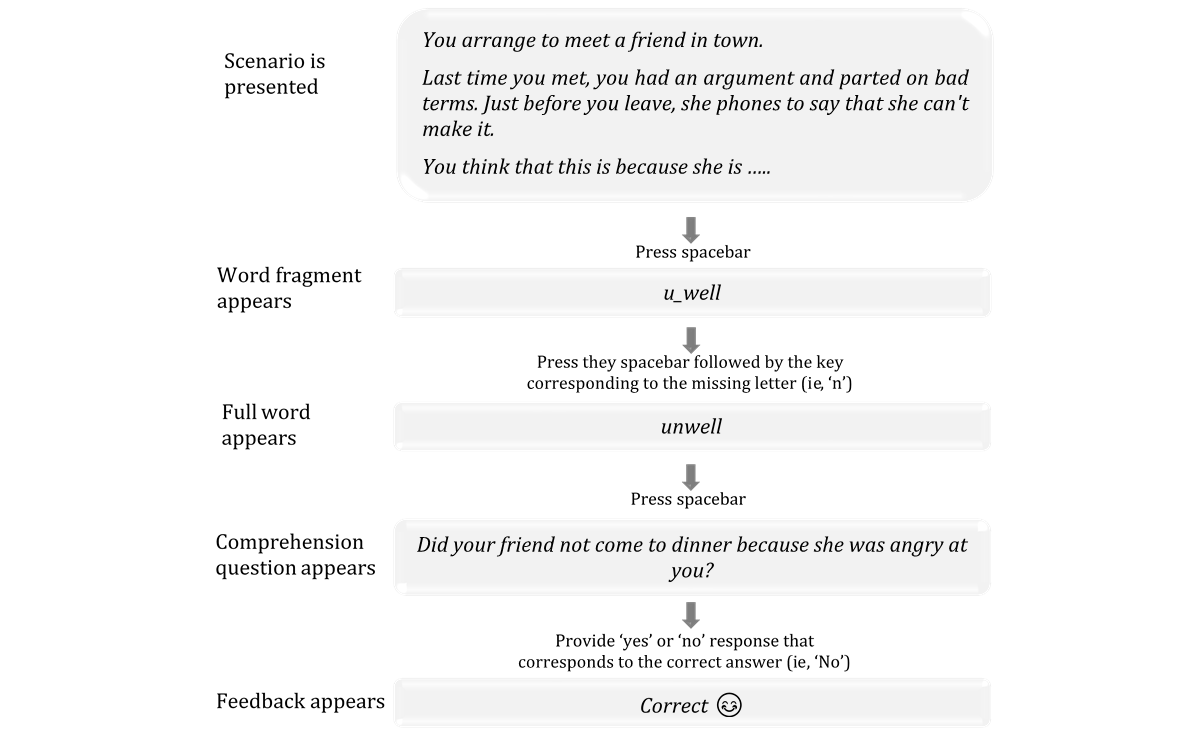
Example of Interpretation Bias Modification scenario to illustrate the training.

In IBM training, participants are trained to resolve ambiguous social scenarios with either positive or benign outcomes through the completion of a word fragment (Figure 3) [22]. Each scenario consists of three lines that are ambiguous in terms of valence or emotional interpretation. The participants are instructed to imagine or visualize themselves in each situation described. One word of the story is presented as a word fragment, which disambiguates the story in a positive or benign way. Participants are asked to complete the fragment as quickly as possible by pressing the spacebar when they know what the word is and then to press the key corresponding to the missing letter. The reaction times are recorded. The program continues only when a correct response is provided. After this, a comprehension question appears that reinforces the assigned meaning when the word fragment is completed. Participants answer the question with a *Yes* or *No* response. They subsequently receive feedback (a *correct* or *wrong* answer, with a corresponding smiley or sad face emoji) to reinforce the interpretation imposed by the word fragment. Points are awarded for each correct letter (+1) and correct answer to the comprehension question (+1). The social scenarios are translated versions of those used in previous research [22,45,76], adapted for this study’s target age range of 18-30 years and the Australian context [51]. Three practice trials are given at the outset of the IBM training component.

##### Delivery Formats of the Re-train Your Brain ApBM+IBM Intervention

###### Group 1: Integrated ApBM+IBM Intervention, With Biweekly Sessions Combining IBM and ApBM

Group 1 will receive 10 biweekly training sessions containing shortened versions of *both* IBM and ApBM within each session (50:50 ratio) plus TAU. For the ApBM component, participants will be presented with frames containing 10 images of alcoholic beverages and 10 images of nonalcoholic beverages, in a random order. Each alcoholic and nonalcoholic beverage is presented three times, for a total of 60 image presentations. For the IBM component, participants will be presented with three blocks of 10 social scenarios (ie, 30 scenarios per session; 300 across the 10 sessions). Each block will contain eight positive modification (*induction*) scenarios and two probe or irrelevant scenarios that have either a positive or benign outcome, to make the induction less obvious. Blocks are in a fixed order, but the order of the scenarios within blocks is random for each participant.

###### Group 2: Alternating ApBM+IBM Intervention, With Biweekly Sessions Alternating Between IBM and ApBM

Participants in group 2 will receive the same treatment dose as group 1; however, on alternating weeks, participants will receive either ApBM *or* IBM (ie, each session will switch between retraining alcohol or anxiety biases in an alternating pattern; five ApBM and five IBM sessions) plus TAU. For each of the five ApBM training sessions, participants will be presented with 120 (rather than 60) alcoholic and nonalcoholic beverage images (20 alcoholic and 20 nonalcoholic, shown three times in random order), and each of the five IBM sessions will provide 60 scenarios (rather than 30).

#### Control Group (Group 3: TAU Only)

The control group will receive TAU, which will be the model of care provided in accordance with standard practices at their service. The focus of the psychological treatment will not be limited to the provision of treatment for anxiety and/or alcohol use (ie, clients may be receiving treatment for other conditions in addition to anxiety and/or alcohol use). No restrictions will be placed on the type of psychological treatment or how long the client has been receiving the treatment. Details regarding the type, length, and focus of treatment will be ascertained during the baseline and follow-up surveys and reported upon in the trial outcomes (more information given in Multimedia Appendix 1). The decision to have few restrictions tied to the treatment offered as part of TAU follows a desire to test the CBM program in the context of real-world care and to evaluate the adjunctive intervention with a deployment focus. This group will be offered the opportunity to receive the *Re-train Your Brain* program (in whichever format is deemed preferable by participants) after all follow-up surveys and measurements are taken at the 3-month assessment point.

### Procedure

#### Study Procedure

All participants will be directed to the *Re-train Your Brain* website, which contains detailed information about the study and provides a direct link to the online participant information statement and consent form. After providing consent, prospective participants will complete a brief, 10-minute online screening survey to determine eligibility. Eligible participants will gain access to an online baseline survey, which will take approximately 30 minutes to complete. Participants will be asked to provide their name, email, and postal address so that the research team may post them a computer mouse to complete a cognitive assessment (*note*: as these assessments are reaction time based, this will ensure consistency in mouse settings between participants, such as pointer speed, etc) and send them a link to the alcohol approach and interpretation bias cognitive assessment. Immediately following the cognitive assessment, participants will be individually randomized by the study website to receive the following: (1) the *integrated Re-train Your Brain* intervention plus TAU (n=30), (2) the *alternating Re-train Your Brain* intervention TAU (n=30), or (3) TAU only (n=30). Participants in the *Re-train Your Brain* intervention groups will be asked to complete a 30-minute motivational interviewing or psychoeducation-based module, followed by 10 cognitive training sessions, delivered online twice per week for 5 weeks (approximately 2-4 days apart). Participants will be reminded of these training sessions via multiple reminder calls, emails, and SMS text messages. As difficulty and boredom are intrinsically part of these kinds of implicit interventions, participants will also receive 3-4 brief motivational enhancement texts or emails over the 5-week program to normalize any difficulties encountered in the training execution and reaffirm their commitment to change. In addition, to maximize engagement with the research trial, participants in all groups will be asked to complete two weekly 5-minute assessments of their anxiety and alcohol use symptoms, for which they will receive a Aus $5 (US $3.80) e-gift voucher per occasion (10 total), with an accumulated voucher (max Aus $50; US $37.70) paid at the end of the treatment period.

All participants will be emailed a link to complete an online survey and a (separate) cognitive assessment at postintervention (ie, 6 weeks postbaseline, to provide 1 week of flexibility in the rate of training completion) and 3 months postbaseline. The survey and cognitive assessments are expected to take approximately 45-60 minutes (combined) to complete at each time point. Consistent with previous online trials [77,78], participants who complete both the online survey and cognitive assessment will receive an Aus $30 (US $22.60) e-gift voucher at each time point as reimbursement for their time participating in the research. To minimize data attrition, the following evidence-based strategies will be used [79,80]: (1) monetary incentives for each assessment completed, (2) collection of multiple sources of contact information (eg, email, mobile number, and postal address), (3) user-friendly electronic survey design that can be completed via multiple devices (eg, via mobile phone), (4) personalized reminder messages (SMS text messaging and email) to complete surveys or cognitive assessments, and (5) a follow-up letter and telephone call to those participants who do not respond. The intervention and cognitive assessments will be accessed via the study website and run using JavaScript, whereas all online surveys will be delivered via Qualtrics. The trial will be conducted in accordance with the SPIRIT (Standard Protocol Items: Recommendations for Interventional Trials) 2013 Statement and CONSORT (Consolidated Standards of Reporting Trials) guidelines.

#### Randomization

To avoid bias, participants will be individually randomized to the *Re-train Your Brain* integrated group, the *Re-train Your Brain* alternating group, or the control group (on a 1:1:1: basis) via the trial website using a computer-generated randomization sequence, which is concealed from the investigators. This process removes the potential for researcher involvement. Randomization will occur directly after the completion of the online baseline cognitive assessment (Figure 1).

### Statistical Analysis and Power Calculations

As this is a pilot trial, a formal power calculation is not required [81,82]. The pilot trial is essential to determine the expected size of effect, which will be used to inform the sample size of a future definitive RCT aiming to assess the efficacy of the *Re-train Your Brain* program. Rates of attrition will be used to indicate estimated follow-up rates in future trials. On the basis of several rules of thumb used to determine an appropriate sample size for a pilot study [83-85], a sample size of 90 young people (30 per arm) was selected to provide sufficient data on the feasibility, acceptability, and preliminary efficacy of the program, although we recognize that the scope of this pilot trial does not allow for a fully powered test of efficacy. Data will be collated and analyzed using StataCorp data analysis software [86].

Descriptive statistics for primary and secondary outcomes will be conducted based on frequencies and cross-tabulations. Analyses for secondary (preliminary) efficacy outcomes will use multilevel mixed effects analysis for repeated measures, which is a flexible analytic approach for modeling change over time using RCT data [87,88]. All models will use baseline measurements as the reference point to estimate participant-specific starting points and change over time. The intervention condition will be represented by a dummy-coded variable, and the condition by time interaction will be examined to assess between-group differences in outcomes over time. Analyses will be consistent with an intention-to-treat framework, with all randomized participants included in the analysis models, regardless of training completion or loss to follow-up. Missing data will be accommodated in these models using maximum likelihood estimation. Preliminary models will be estimated, with model fit statistics examined to determine the most appropriate model and covariance structure. Cohen *d* will be calculated from model-estimated marginal means and SEs to determine the size of the effect between conditions at the relevant end point. In addition, effect sizes will be analyzed as a function of session completion (ie, completion of six or more training sessions, given that this has been identified as the mean optimum number of ApBM sessions in past research [67]).

## Results

Recruitment is expected to be completed by mid-2022 to late 2022, with the 6-week and 3-month follow-ups to be completed by early 2023. The results are expected to be submitted for publication in 2023. The research team intends to present the findings of this trial at professional seminars and national and international conferences. Only aggregated group data will be reported, and no individuals will be identified.

## Discussion

### Principal Findings

This paper presents the design of the *Re-train Your Brain* study, a randomized controlled pilot trial seeking to evaluate the feasibility, acceptability, and preliminary efficacy of a 10-session, web-based ApBM+IBM brain training program for hazardous alcohol use and social anxiety among young Australians aged 18-30 years, when combined with TAU. To the best of our knowledge, this is the first study to evaluate a comorbidity-targeted intervention of this kind, using a cost-effective, web-based delivery method.

### Strengths and Limitations

This study has several strengths. First, the intervention was co-designed with emerging adults who have direct lived experience of hazardous drinking and social anxiety and clinicians who have experience treating anxiety and/or alcohol use concerns. This bottom-up approach, whereby emerging adults and clinicians were involved in the development of the methodology and stimuli used within the intervention (eg, the alcoholic or nonalcoholic beverages used in ApBM and the scenarios included in IBM), was critical to ensuring that the program is relevant, engaging, and useful for end users. This codevelopment process also helps to ensure that the program can be feasibly implemented and is responsive to the needs of service providers.

An additional strength of this study is the web-based delivery of the interventions. The *Re-train Your Brain* intervention is simple, inexpensive, and can be self-administered via the internet without any specific skills or knowledge. This format allows for easy and convenient access anywhere and at any time (eg, in the privacy of one’s home), thereby offering a larger outreach and greater availability. It is also being investigated in the setting in which it is most likely to be implemented, if proven effective in a future RCT. On theoretical and practical grounds, incorporation of a web-based psychoeducational or motivational enhancement-type module at the outset of training will aid its efficacy and will also likely boost engagement and user buy-in. It may also increase treatment adherence and reduce study attrition. Thus, the web-based delivery of this program has the potential to enhance clinical outcomes at a minimum cost in terms of time and effort for both patients and service providers.

Finally, despite their high co-occurrence, to date, most CBM programs have addressed anxiety and alcohol use in isolation of one another, with a few exceptions for attention bas modification [48]. The current intervention is unique in that it combines effective alcohol ApBM and anxiety IBM-focused protocols to optimize standard treatments among a young comorbid sample. Given the interconnections between anxiety and alcohol use problems, whereby each disorder fuels the other and impedes recovery from the other, it is possible that combining these programs will have incremental (or synergistic) effects on anxiety and alcohol use outcomes in a vulnerable group that responds poorly to standard treatments [10,89].

One of the main strengths of this study is also its main limitation: that is, its web-based delivery. First, internet interventions are conducted in a less-controlled home environment that may pose several online distractions (eg, email notifications) and offline distractions (eg, housemates or phone calls), which have the potential to impact the study results. Second, web-based interventions also have higher rates of attrition compared with standard face-to-face treatments (eg, 34.2% vs 24.6%, respectively) [90]. Further increasing the risk of attrition, CBM interventions are inherently repetitive and as such are sometimes considered boring because of the monotonous nature of the tasks [28,91]. To help overcome these limitations, at the start of each session, participants are reminded of the importance of conducting training in a quiet environment where they can concentrate for a 20-minute block. To leverage on participants’ intrinsic motivation for change and maximize their initial buy-in, a compelling rationale and motivational enhancement module is provided at the outset of the program. These components will set expectations about the purpose and nature of the intervention (eg, computerized and repetitive training), which will likely enhance motivation to train and, thus, result in increased adherence and lower attrition. In addition, multiple evidence-based strategies to minimize treatment and study attrition will be used, such as monetary incentives, email and SMS text messaging reminders, and explanation of the importance of completing the sessions and follow-up surveys to participants [79,80]. Taken together, the possible threats presented by the web-based delivery of the intervention are outweighed by the possibilities and benefits it may offer.

Furthermore, this study uses a control group that receives TAU and zero training, rather than a control group that is matched for both stimulus exposure (ie, time spent completing the ApBM and IBM tasks) and response requirements. Sham control-training groups are considered the optimal comparator groups for these reasons and because participants remain blind to their group allocation [92]. However, some research has shown that there is no significant difference between experimental and sham control groups (as both groups improve [33,92]), suggesting that sham training may in fact have an active component rather than it being a *neutral* or *placebo* training it is intended to be. This is particularly the case for online trials and less so for laboratory-based trials [47]. This way, it is conceivable that both online-delivered sham and experimental conditions represent placebo effects; however, this cannot be ruled out in a two-arm design (active vs control training). Given that this study is a pilot trial with primary outcomes focused on feasibility and acceptability (as opposed to efficacy) and secondary outcomes on preliminary efficacy, an active control group could mask any possible intervention effects, and thus, a no-training group was selected as the comparator. Indeed, in this study, priority was given to testing two experimental varieties on top of TAU, as compared with TAU. Future RCTs aiming to evaluate the clinical efficacy of this hybrid intervention may consider a different three-arm design, comparing the ApBM+IBM intervention against both an (active) sham-training control and a zero training control condition (ie, TAU), and examine potential mediation effects across the three conditions.

### Conclusions and Implications

This world’s first hybrid ApBM+IBM training program combines the best elements of efficacious ApBM training programs for alcohol use problems and IBM for social anxiety into a hybrid program for emerging adults who experience both of these problems. This innovative program can be delivered over the internet and can thereby maximize efficiency and scarce resources and sustainably increase the intervention options for vulnerable populations at a low cost. Given the costs of conducting an RCT, it is important to establish whether the *Re-train Your Brain* ApBM+IBM program is acceptable to emerging adults and whether it is feasible to deliver via the internet, in settings where it will ultimately be used and easily scaled to, including at home. The pilot trial findings will contribute to understanding the types of programs that emerging adults will engage with and whether there are signs of it being an efficacious treatment option for this comorbidity.

## Appendix

Multimedia Appendix 1 A detailed description and interpretation of scores for primary and secondary outcome measures.

## Abbreviations

ApBM: approach bias modification
CBM: cognitive bias modification
CONSORT: Consolidated Standards of Reporting Trials
IBM: interpretation bias modification
RCT: randomized controlled trial
SPIRIT: Standard Protocol Items: Recommendations for Interventional Trials
TAU: treatment as usual
