# Supplementary material for: A Web-Based Cognitive Bias Modification Intervention (Re-train Your Brain) for Emerging Adults With Co-occurring Social Anxiety and Hazardous Alcohol Use: Protocol for a Multiarm Randomized Controlled Pilot Trial
Source: JMIR Res Protoc. 2021 Jul 7;10(7):e28667. doi: 10.2196/28667 (PMC8295835; doi:10.2196/28667)
Supplement: Multimedia Appendix 1 [file resprot_v10i7e28667_app1.docx]

#### Multimedia Appendix 1

#### Adverse events

Assessment of adverse events will follow the consensus statement for defining and measuring negative effects in internet interventions [57]. Serious adverse events will be defined as: suicide attempts, death, and hospitalisation. Adverse events will be defined as deterioration of social anxiety symptoms or alcohol use, according to the widely used reliable change index [58]. Three methods will be used to assess any adverse events associated with treatment:

1. spontaneous reports in open feedback questions probing about the experience of negative effects during treatment,
2. spontaneous reports to the study team via email, SMS, or phone in response to study prompts and reminders,
3. deterioration in social anxiety (SIAS-6 and SPS-6) or alcohol use (TLFB standard drinks per day) from pre- to post-assessment, determined according to the widely used reliable change index [58].

All spontaneously reported incidents will be recorded, and the participant’s attribution of the deterioration will be coded as being related or unrelated to the *Re-Train Your Brain* program.

#### Interpretation and expectancy biases

*Interpretation and expectancy biases for co-occurring social anxiety and alcohol use* will be assessed by the self-report Comorbid Social Anxiety and Alcohol Interpretation Bias task [64]. Participants are presented with a set of eight ambiguous scenarios related to: drinking to manage a social situation, drinking to reduce shame after a social situation, and embarrassment after a heavy drinking episode. Each scenario is followed by three possible explanations for the situation. One of the explanations for each scenario is associated with a link between social concerns and alcohol use (e.g., “You want a drink so you will have an easier time talking to people”), whereas the other responses are not (e.g., *“*You are thirsty and want to grab a soda.”). Participants will be asked to rate the degree to which each of the three explanations would likely be true if they were in that situation (0 ‘not at all likely’ to 8 ‘extremely’). The item score for the comorbidity-relevant response options is averaged across the set of scenarios.

#### Alcohol use

*Alcohol consumption* (average drinks per day) and frequency of *binge drinking* days (>5 standard drinks per drinking day) in the past month will be assessed through a computerised version of the Timeline Follow-back Procedure [65-67]. *Hazardous alcohol use* will be assessed through the 10-item AUDIT [54]. Total scores range from 0–40, with cut-off scores of 8–15, 16–25, and ≥26 indicating risky/hazardous alcohol use, high risk/harmful use, and high risk/possible dependency. *Severity of alcohol dependence* will be assessed by the 20-item Severity of Alcohol Dependence Questionnaire [68]. Each item is scored from 0 ‘almost never’ to 3 ‘nearly always’, with total scores ranging from 0 to 60. Higher scores indicate a greater dependence for alcohol. *Alcohol cravings* will be assessed by the 12-item Severity of Alcohol Craving Questionnaire–Short Form–Revised. Each item is scored from 1 being ‘strongly disagree’ to 7 being ‘strongly agree’ with higher scores indicating stronger cravings for alcohol. *Motives for alcohol use* in the past month will be assessed through the 28-item Drinking Motives Questionnaire-Revised [69]*.* Participants are asked to indicate how frequently they drink alcohol for a range of reasons (grouped into five subscales: coping-anxiety, coping-depression, social, enhancement, and conformity), scored on a 5-point Likert scale ranging from 0 ‘almost never/never’ to 4 ‘almost always/always’. Total scores range from 0 to 40, with responses averaged for each subscale. To assess *alcohol use* throughout the intervention period, all groups will be asked to complete the past week Timeline Follow Back [65-67]. This will enable an accurate assessment of participants’ frequency of drinking, drinks per drinking day, and frequency of binge drinking (5 or more standard drinks) each week.

#### Anxiety

*Symptoms of social anxiety*, including interaction and performance fears, will be assessed using the Social Interaction Anxiety Scale (SIAS-6) and Social Phobia Scale-short forms (SPS) [55]. The 12 items of the SIAS-6 and SPS-6 are scored on a 5-point Likert scale, ranging from 0 being ‘not at all true or characteristic of me’ to 4 being ‘extremely true or characteristic of me’. Total scores range from 0-48, with cut-off scores of ≥7 on the SIAS-6 and ≥2 on the SPS-6 being used to indicate a possible diagnosis of social phobia (used for inclusion in current study). *Symptoms of depression and anxiety* will be assessed by the 4-item Patient Health Questionnaire-4 [70]; an ultra-brief screener which combines a two-item measure for depression (PHQ–2), as well as a two-item measure for anxiety (Generalized Anxiety Disorder–2). The items are scored on a 4-point Likert scale, ranging from 0 ‘not at all’ to 3 ‘nearly every day’. Total scores for each subscale range from 0 to 6, with a score of 3 or greater on either the depression or anxiety subscale representing a reasonable cut-point for identifying potential cases of major depression and anxiety (generalized anxiety, panic, social anxiety, and posttraumatic stress disorders), respectively To assess change in social anxiety symptoms throughout the intervention period, all groups will be asked to complete the Social Phobia Weekly Summary Scale [71] on a weekly basis. The measure consists of six items that assess the severity of social anxiety, social avoidance, self-focused versus external attention (in general and in difficult social situations)*,* anticipatory processing, and post-event rumination over the past week. Each item is rated on a Likert-type scale ranging from 0 to 8, with higher scores corresponding to greater social anxiety symptomology.

#### Psychological and pharmacological treatment

Participants will be asked whether they have received any treatment from a health professional for anxiety symptoms or alcohol use problems in the past 3 months (yes/no), who they consulted, they type of professional they consulted (i.e., counsellor, psychologist, clinical psychologist, GP, psychiatrist, hospital inpatient admissions, inpatient or residential treatment, emergency department visits, other), what they consulted them for (anxiety, drinking, other), whether treatment was current (yes/no) and what type of treatment they received (cognitive behavioural therapy, counselling, alcohol/other drug counselling, dialectical behavioural therapy, acceptance & commitment therapy, motivational interviewing, schema therapy, other, unsure). Participants will also be asked whether they have taken any medication for anxiety, depression, or alcohol use problems, what the medication was, when they started/ceased taking them, and whether medications were current (yes/no).
